# Silicon Carbide Nanoparticles as a Mechanical Boosting Agent in Material Extrusion 3D-Printed Polycarbonate

**DOI:** 10.3390/polym14173492

**Published:** 2022-08-26

**Authors:** Markos Petousis, Nectarios Vidakis, Nikolaos Mountakis, Sotirios Grammatikos, Vassilis Papadakis, Constantine N. David, Amalia Moutsopoulou, Subrata C. Das

**Affiliations:** 1 Department of Mechanical Engineering, Hellenic Mediterranean University, 71410 Heraklion, Greece; 2 Laboratory for Advanced and Sustainable Engineering Materials (ASEMlab), Department of Manufacturing and Civil Engineering, Norwegian University of Science and Technology, 2815 Gjovik, Norway; 3 Institute of Molecular Biology and Biotechnology, Foundation for Research and Technology—Hellas, 71110 Heraklion, Greece; 4 Manufacturing Technology & Production Systems Laboratory, School of Engineering, International Hellenic University, Serres Campus, 62124 Serres, Greece

**Keywords:** three-dimensional printing, fused filament fabrication, nanocomposites, polycarbonate, silicon carbide, mechanical characterization

## Abstract

In this work, the effect of silicon carbide (carborundum, SiC), as a boosting agent of the mechanical response of the polycarbonate (PC) polymer, was investigated. The work aimed to fabricate nanocomposites with an improved mechanical performance and to further expand the utilization of 3D printing in fields requiring an enhanced material response. The nanocomposites were produced by a thermomechanical process in various SiC concentrations in order to evaluate the filler loading in the mechanical enhancement. The samples were 3D printed with the material extrusion (MEX) method. Their mechanical performance was characterized, following international standards, by using dynamic mechanical analysis (DMA) and tensile, flexural, and Charpy’s impact tests. The microhardness of the samples was also measured. The morphological characteristics were examined, and Raman spectra revealed their structure. It was found that SiC can improve the mechanical performance of the PC thermoplastic. A 19.5% increase in the tensile strength was found for the 2 wt.% loading nanocomposite, while the 3 wt.% nanocomposite showed a 16% increase in the flexural strength and a 35.9% higher impact strength when compared to the unfilled PC. No processability issues were faced for the filler loadings that have been studied here.

## 1. Introduction

Thermoplastics are in increasing demand in various industries, such as automotive, medical, and electronics, because they are lightweight materials that are easy to process and they are cost-effective [1,2]. Polycarbonate (PC) is a popular thermoplastic that is used in various everyday applications, such as packaging and consumer goods [3], but also in advanced applications, such as nanoelectronics [4], sensors [5], and laser applications [6]. In the medical field, PC has been used in orthopedics [7], in stents [8], and in antibacterial applications [9]. Therefore, in the literature, it has been thoroughly studied for its mechanical (dynamic loading [10], tensile response [2], creep [11], and hardness [1]) and thermal properties [2,10], in pure form or as a matrix material in composites, with fillers, such as carbon fillers or carbon nanotubes [2,10,12,13]. In material extrusion (MEX), 3D printing (3DP), in which the fused filament fabrication (FFF) process belongs, the parts are built with thermoplastics. In this process, polylactic acid (PLA) [14] is the most popular thermoplastic that is used. Thermoplastics are used as pure or as matrix materials, for the development of composites and nanocomposites, in aiming to study their performance and to improve their properties [15,16,17,18,19,20,21]. The PC polymer has been thoroughly investigated in the MEX 3DP process for its mechanical properties [22] and various other aspects of its performance, which are presented further below. The impact of the parameters that are utilized in the 3DP process on the mechanical response of the PC polymer have also been investigated and optimized with statistical modeling tools [23,24,25,26]. The flow of the material during the MEX process has been investigated with numerical modeling tools [27] and the effect of weathering on the mechanical properties of the parts that are made with additive manufacturing (AM) has been studied. It was reported that weathering has a significant effect on the mechanical properties [28]. The thermal properties of the 3DP PC samples have also been reported in the literature [29,30]. PC has been used as a matrix material in MEX 3DP for the development of composites, aiming to improve its performance and to provide multi-functional characteristics to the developed materials [29,31,32,33]. PC in 3DP is used for medical [34,35,36,37] and acoustic applications [38], among others, and it has been proven to be an eco-friendly biopolymer for indoor applications [39].

Silicon carbide (SiC) is a robust ceramic material [40], and one of the hardest materials available [41]. Due to its excellent properties (oxidation resistance [40], hardness [41], high temperature resistance [42], and high strength [43]), it has been utilized in different applications, such as nuclear [40,44], batteries [45], optics [46,47], photonics [48], high temperature applications [42], semiconductors and electronic devices [49,50,51,52], coatings [53], friction, defense applications [43,54], healthcare applications in medical implants and devices [41,55], and humidity sensors [56]. SiC is employed as a filler in composites and nanocomposites in order to induce specific properties in the materials and to be able to confront the requirements of demanding applications. The performance of these materials has been presented and investigated in the literature, e.g., high corrosion resistance [56], low cost, flexibility, and antibacterial activity [56], mechanical properties enhancement and thermal stabilization [57], improved mechanical response [58], enhanced thermal conductivity [59], and enhanced mechanical, thermal properties, and moisture absorption [60]. Due to the wide field of applications, it has potential for use in AM applications; however, the literature in this field is still very limited. SiC in AM has been investigated in the selective laser sintering (SLS) process [44,54,61,62,63,64] and the binder jetting AM [65,66]. To the authors’ knowledge, no literature is currently available employing SiC as a filler in MEX 3DP. Regarding the use of SiC as a filler in composites using PC as the matrix material, research focuses mainly on the development and investigation of polymer blends [67,68,69]. To the authors’ knowledge, only one study has reported on PC/SiC nanocomposites in the literature, with the subject of the work not including the mechanical response of the prepared materials, which would be useful in the current work for evaluation and comparison purposes [70]. None of these works that are presented in the literature regarding the PC polymer and SiC are related to the AM technology.

In this study, for the first time SiC, in nanopowder form, was introduced as an additive in the PC thermoplastic and nanocomposites were prepared and investigated in MEX 3DP. The nanocomposites were fabricated at different filler loadings in order to study the impact of the additive concentration as a boosting agent of mechanical performance. The feasibility of the process was evaluated, aiming to present materials with enhanced mechanical properties for the 3DP process, exploiting its benefits, and expanding its fields of application. Such enhanced mechanical properties are crucial for the design of the parts, since it leads to the decrease in the mass of the parts and their size and, therefore, to the reduction in required materials. The nanocomposites herein were produced utilizing a thermomechanical procedure. The samples were then 3D printed with the MEX process and were subjected to mechanical testing, following the corresponding international standards. Raman’s analysis was performed in order to investigate the structure of the materials. The morphology of the produced filament was examined with atomic force microscopy (AFM), while on the 3DP samples, scanning electron microscopy (SEM) was employed. The thermal properties of the prepared nanocomposites were determined with thermogravimetric analysis (TGA) and differential scanning calorimetry (DSC). Overall, for filler loadings up to 3 wt.%, SiC improved the mechanical response of the material when compared to the unfilled PC thermoplastic. This verifies that SiC can serve as a boosting agent in MEX 3DP for the polymer studied herein (PC). Moreover, inducing the SiC additive in nanoscale form into the PC polymer did not compromise the matrix material’s processability or thermal stability. Considering the numerous applications where SiC is used, such results show the potential of using it as a filler in 3DP.

## 2. Materials and Methods

### 2.1. Materials

PC of type EMERGE 8430-15 was the matrix material used in this study (tensile strength of 70.0 MPa, density of 1.20 g/cm^3^). It was procured from Styron Europe GmbH (Styron Europe GmbH, Horgen, Switzerland) in powder form. The filler of the study (SiC) in nanopowder form, was sourced directly from its manufacturer (Nanographi, Ankara, Turkey), having the following specifications: a cubic shape, true density of 0.05 g/cm^3^, purity of 99.5+%, specific surface area of 40–85 m^2^/g, size of 50–70 nm, and melting point of 2700 °C.

### 2.2. Production of the Nanocomposites in Filament Form

**Figure 1:**
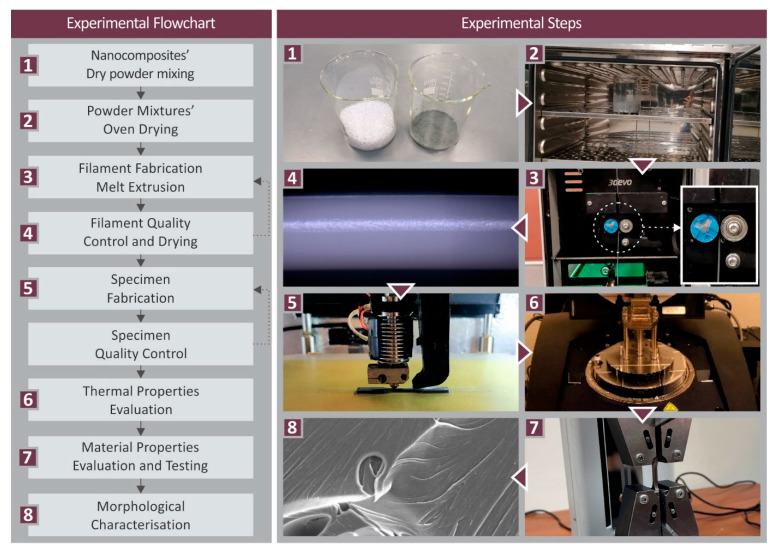
Methodology of the current work.

The methodology followed in the work for the preparation and the characterization of the PC/SiC nanocomposites is presented in Figure 1.

The raw materials were used to produce a filament that was compatible with the MEX 3DP process (1.75 mm in diameter), with a thermomechanical process successively repeated twice, to achieve good additive dispersion in the matrix. Different mixtures (four) of the matrix material and the filler were made, at different weight-to-weight concentrations (filler loading of 1, 2, 3, and 6 wt.%), to produce nanomaterials with the extrusion process, with corresponding filler loadings. Each material’s mixture was vigorously mixed for 30 min in a high-power blender. To prevent the powder from spreading throughout the air of the room, the procedure was carried out in a glovebox. Any moisture in the raw materials was initially removed, following a drying process in a laboratory oven (24 h, 60 °C). The drying process was conducted twice, before and after the mixture of the raw materials. The mixtures were then successively fed into a Noztek (Noztek, Shoreham-by-Sea, UK) extruder (featuring a single screw) and the corresponding nanocomposites were produced in filament form. These filaments were shredded into pellets (3devo shredder, Utrecht, The Netherlands). The second step of the thermomechanical process for the nanocomposites production was to convert these pellets again into a filament that was compatible (1.75 mm diameter) with MEX 3D printers (3devo Composer, Utrecht, The Netherlands). In the second step of the process, the extruder used had a special geometry screw for material mixing. The parameters set on the extruder were a screw speed of 4.8 rpm and temperatures set at 200 °C at positions 1–3, and 240 °C at the fourth heating zone (near the hopper). These parameters were determined with preliminary experiments conducted prior to the fabrication of the current work’s filaments. Pure PC filament was also prepared to be used for evaluation purposes.

### 2.3. 3D Printing of the Samples for Mechanical Testing

**Figure 2:**
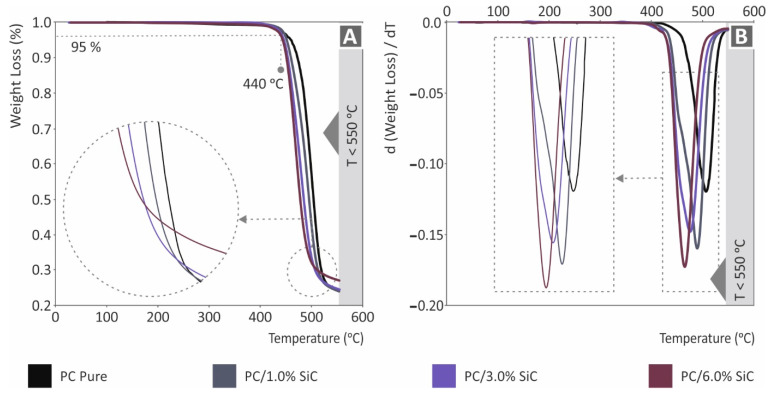
Pure PC and PC/SiC nanocomposites TGA results: (**A**) graph of the weight loss (%) compared to temperature (°C); (**B**) graph for the mass degradation rate (dw/dT) compared to temperature (°C).

The PC/SiC nanocomposites produced with various SiC concentrations in filament form were utilized to fabricate MEX 3DP specimens, in accordance with the corresponding international standards (Figure 2), for the mechanical response characterization of the materials. Five specimens were fabricated from each different material and mechanical test, on an Intamsys, model Funmat HT (Intamsys, Shanghai, China) MEX 3D printer. The required G-codes were compiled utilizing the Intamsuite software v3.8.0 (Intamsys, Shanghai, China). The 3DP parameters were determined with preliminary experiments conducted prior to the fabrication of the current work’s samples. A ±45 degrees line infill pattern was used. The layer thickness was set to 0.2 mm, 3D printer bed temperature was set to 115 °C, nozzle temperature was set to 260 °C, and chamber temperature was set to 65 °C. Two perimeters were built, along with four solid layers on the top and the bottom of the parts. The infill density was set to 100% and the 3D-printing speed was set to 25 mm/s (please see also Supplementary Material Figure S1).

### 2.4. Nanocomposites Thermal Properties and Structural Analysis

To identify the thermal stability of the produced nanocomposites, and their thermal properties in general, thermogravimetric analysis (TGA) (Perkin Elmer Diamond, Waltham, MA, USA, 40–550 °C, 10 °C/min step, a nitrogen atmosphere) and differential scanning calorimetry (DSC) (TA Instruments 25, New Castle, DE, USA, 25–225 (5 min) −25 °C, 15 °C/min step) were employed. 

Raman spectroscopy was conducted by a modified LabRAM HR Raman spectrometer (HORIBA Scientific, Kyoto, Japan). A solid-state laser module at 532 nm central wavelength was used, with a maximum laser output power of 90 mW. A 50× microscopic objective lens with a 0.5 mm and a long working distance of 10.6 mm numerical aperture (LMPlanFL N, Olympus, Tokyo, Japan) delivered the excitation light and collected the Raman signals. The laser spot dimensions were approximately 1.7 μm of spot diameter and about 2 μm of axial focal length. A neutral density (ND) filter was used that allowed 5% of laser light to go through, which resulted in 2 mW power on the sample. The Raman spectral resolution was ~2 cm^−1^ using a 600 groves grating. The acquired Raman spectral range was set from 300 to 3100 cm^−1^, resulting in 2 optical windows or 2 acquisitions per point. For each measurement, 10 seconds of acquisition time and 5 accumulations were used. All Raman spectra were processed with LabSpec 6 (HORIBA Scientific, Kyoto, Japan). Initially, the background was removed utilizing an internal function operation utilizing a polynomial fit. Data acquired on the measurements were normalized utilizing unit vector, thus achieving increased comparison levels between the samples.

### 2.5. Evaluation of the Nanocomposites’ Filament Quality and Mechanical Performance

The filament produced before the fabrication of the 3DP specimens was examined for its morphological characteristics and its mechanical strength. The diameter was monitored during its production on the 3devo Composer (Utrecht, The Netherlands) extruder, exploiting the real-time diameter-measuring sensor of the extruder. Afterward, it was measured with a high-quality caliper in random positions to evaluate the automatic measurements and to ensure that its diameter was compatible with the 3DP process requirements. Additionally, its side surface morphological characteristics were investigated with atomic force microscopy (AFM) (MicroscopeSolver P47H Pro, Moscow, Russia, 300 kHz) to identify the impact of the additive introduction on the surface quality, which affects the processability of the material. The mechanical performance of the nanocomposites at this stage was evaluated with tensile testing on an Imada MX2 (Northbrook, IL, USA), utilizing a customized special fixture to fix the filaments in the machine. The results of these tests, although they were not following an international standard, due to the geometry of the samples, provided a preliminary indication of the performance of the nanocomposites. Additionally, they can be correlated with the 3DP specimens’ mechanical test results to analyze the impact of the 3DP procedure on the mechanical performance of the nanocomposites in a qualitative manner.

### 2.6. 3DP Specimens’ Mechanical Strength Characterization

For the mechanical characterization of the fabricated 3DP specimens with the produced nanocomposites in the study, five different mechanical tests were conducted, following the corresponding standards, as follows:

- Dynamic mechanical analysis (DMA): Three-point-bending, 30–200 °C, step 5 °C/min, and a magnitude of 30 μm oscillation was set, utilizing a frequency of 1 Hz, and applying a preload of 0.1 N, following the ASTM D4065-12 standard, on a TA Instruments DHR 20 (New Castle, DE, USA) apparatus, prismatic specimen 122 mm × 12.7 mm;
- Tensile test: A Type V specimen was utilized (dogbone with 6.5 mm × 10 mm × 3.2 mm thickness), setting the elongation speed at 10 mm/min, according to the ASTM D638-02a standard, on an Imada model MX2 (Northbrook, IL, USA) apparatus;
- Flexural test: A three-point-bending test was carried out, with a span of 52 mm. Tests were conducted with an elongation speed of 10 mm/min, according to the ASTM D790 standard, on an Imada model MX2 (Northbrook, IL, USA) apparatus, prismatic specimen 64 mm × 12.7 mm × 3.2 mm thickness;
- Impact test: The type of test was Charpy notched. In all experiments, the release height was selected to be 367 mm, following the ASTM D6110, on a Terco MT 220 (Kungens Kurva, Sweden) device, prismatic notched specimen 122 mm × 12.7 mm × 5 mm thickness;
- Microhardness measurements: The type of the measurements accorded to the Vickers method, applying a load of 200 gF, for an indentation time of 10 s, following the ASTM E384-17 standard, on an Innova Test model 300 (Maastricht, The Netherlands) apparatus.

### 2.7. Examination of the Morphological Characteristics of the 3DP Specimens

The morphological characteristics of the 3DP samples were examined with scanning electron microscopy (SEM) (JEOL JSM 6362LV, Peabody, MA, USA, 20 kV, high-vacuum mode, Au-sputtered samples, detector mode SE). To evaluate the quality of the 3DP process, images were acquired from the side surface of the samples. To reveal the fracture mechanism in the tensile tests, images were also acquired from the fracture area of tensile test samples. On un-sputtered specimens, energy-dispersive X-ray analysis (EDX) was also carried out to confirm the chemical composition of the nanocomposites.

## 3. Results and Discussion

### 3.1. Nanocomposites Thermal Properties and Structural Analysis

**Figure 3:**
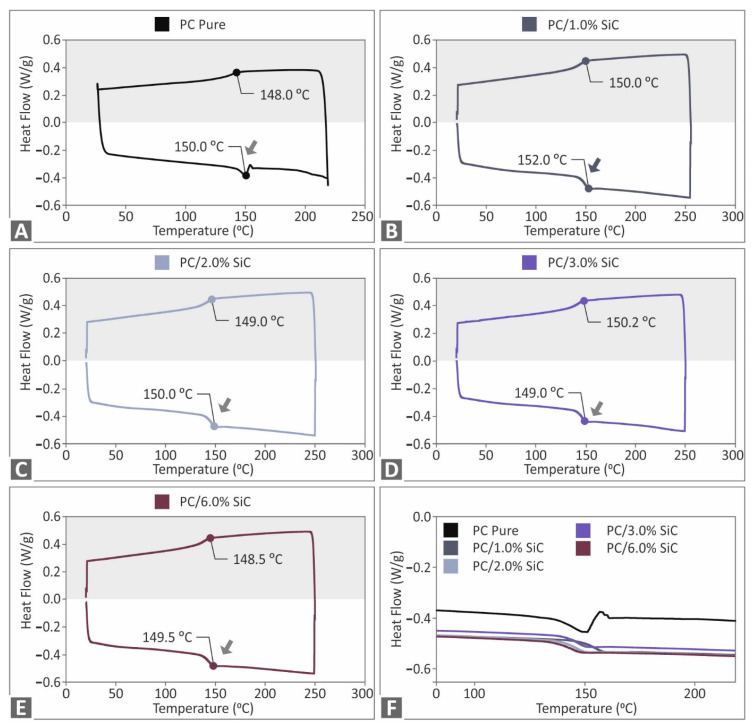
Pure PC and PC/SiC nanocomposites DSC results (endotherm and exotherm heat flow graph, W/g, compared to temperature, °C) for PC: (**A**) pure, (**B**) SiC 1 wt.%, (**C**) SiC 2 wt.%, (**D**) SiC 3 wt.%, (**E**) SiC 6 wt.%, and (**F**) zoom on the exotherm graphs for comparison purposes.

The TGA measurements that were acquired (pure PC, PC/SiC nanocomposites) are presented in Figure 2. All of the materials that were assessed showed no significant degradation up to 440 °C, where an intense weight loss starts to occur. In addition, no significant differences were found between the materials. The addition of the SiC filler shifts the degradation curve to lower temperatures, with an increasing trend, as the loading increases. Still, the differences are not significant between the materials. Figure 2B presents the weight loss rate curves. The introduction of the SiC additive increases the weight loss rate, compared to the unfilled PC thermoplastic. The 6 wt.% nanocomposites (highest loading studies) showed the highest weight loss rate, with an almost 40% increase compared to the pure PC. The highest rate of weight loss is reported at a slightly lower temperature, and a similar behavior with the weight loss curve is depicted. Overall, the temperature of the maximum weight loss rate reduces as the filler loading increases. Figure 3 depicts the DSC results. For the pure PC and the PC/SiC nanocomposites, the differences recorded are not significant, with the characteristic temperatures in the endotherm and the exotherm curves being similar between the different materials. The addition in the pure PC polymer of the additive (SiC) increased the absorbed energy in both the endotherm and exotherm cases, still, the difference between the nanocomposites is negligible, showing that the filler loading does not influence the absorbed energy during the measurements.

The thermal properties in the work showed no significant impact on the PC polymer’s thermal stability with the addition of the SiC filler. Moreover, it was ensured that, for the extrusion and the 3DP process, the nanocomposites were not degraded by the temperatures that were used. The dispersion of the filler (SiC) in the PC thermoplastic was sufficient, with the methodology followed for the preparation of the nanocomposites since, in the SEM images, the agglomerations were not located. In the conducted mechanical tests, the calculated deviation for the mechanical properties was within acceptable limits.

**Figure 4:**
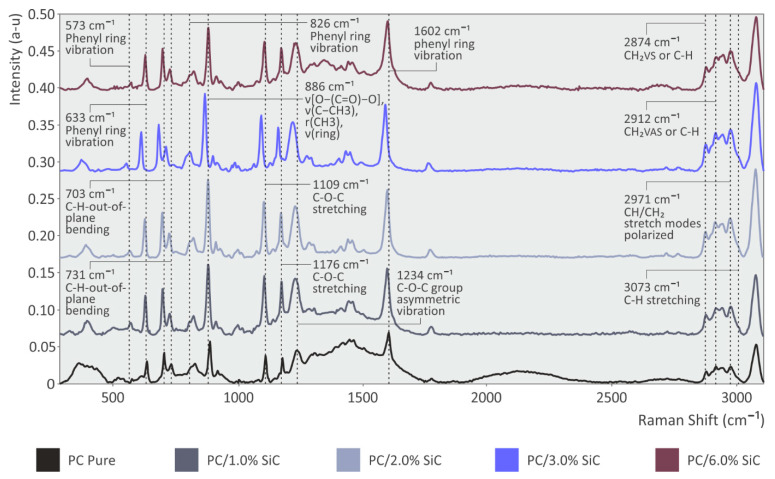
Pure PC and PC/SiC nanocomposites Raman spectra results.

The spectrographic analysis of the unfilled PC and the PC/SiC nanocomposites are presented in Figure 4. Spectroscopy (RS) was performed in order to check the material at the molecular level. As can be seen from the figures, the major contribution comes from the main material PC. In all of the cases, together with the increase in concentration percentage, the photoluminescence signal also increased. Some differences were observed between the different concentrations of SiC within the PC nanocomposite materials. The primary Raman peaks from the analysis of pure PC are shown in Figure 4, along with their associated assignments. The discovered Raman peaks have a range of 573 cm^−1^ to 3073 cm^−1^. The measured spectrum matches the polycarbonate from which the assignments are obtained, according to the literature [71,72,73,74].

Comparing the nanocomposite samples with the pure PC thermoplastic shows that the following Raman peaks that are presented in Table 1 are increased in the PC/SiC specimens. In particular, an increase in all of the SiC samples can be found in the antisymmetric stretching of the Si–O bond at 966 cm^−1^, the C–H bending at 1003 cm^−1^, and the C–O–C stretching at 1175 cm^−1^. Two additional Raman lines present an increase, one at 781 cm^−1^ and the second at 1344 cm^−1^, both of which are related to Si–O stretching.

### 3.2. Evaluation of the Nanocomposites’ Filament Quality and Mechanical Performance

The filament diameter measurements were taken during the extrusion process (please see Supplementary Material Figure S2). The measurements indicate that the diameter of the produced filament is compatible with the 3DP process. In the filament tensile test results, it was shown that the addition of the filler has a positive effect on the tensile strength of the filament (please see Supplementary Material Figure S2). The PC/SiC 1 wt.% filament had the highest improvement (7.6%) in the tensile strength when correlating the results with the unfilled PC material. It also exhibited a stiffer response of 7.8%. The PC/SiC 2 wt.% also exhibited enhanced mechanical properties when correlating the results with the unfilled PC material. At higher loadings, the mechanical response decreases, with the 3 wt.% loading having a similar performance to the pure PC and the 6 wt.% showing an intense decrease in its mechanical response. This indicates that a saturation threshold of the SiC additive in the PC thermoplastic is expected when slightly increasing the filler further than 6 wt.%.

The morphology of the side surface of the filaments that has been produced in this work (pure PC and PC/SiC nanocomposites) was captured with AFM. The inclusion of the SiC additive in the matrix material (PC polymer) increases the surface roughness of the filaments when correlating the results with the unfilled PC polymer. No clear trend can be observed for the surface roughness compared with the filler loading (please see Supplementary Material Figure S3).

### 3.3. 3DP Specimens’ Mechanical Strength Characterization

**Figure 5:**
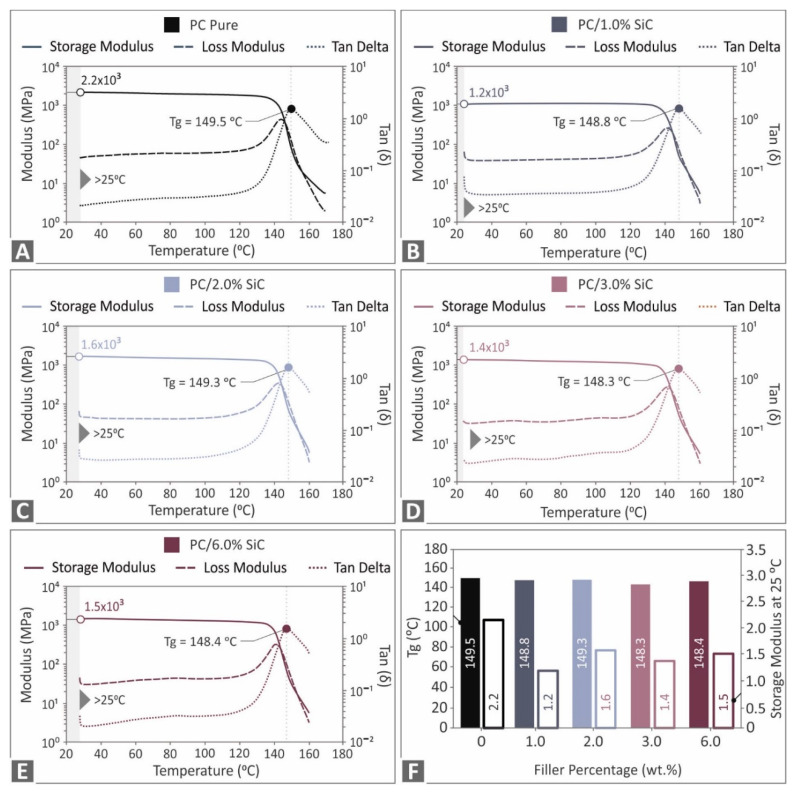
DMA tests results (storage modulus, loss modulus, and tan (delta)), PC: (**A**) pure, and nanocomposites with SiC at loadings of (**B**) 1 wt.%, (**C**) 2 wt.%, (**D**) 3 wt.%, (**E**) 6 wt.%, and (**F**) glass transition temperature Tg (°C) at 25 °C, and the corresponding storage modulus values.

The graphs of the storage modulus, the loss modulus, and the tan (delta) that were obtained from the DMA experiments for the pure PC and the nanocomposites with the SiC concentrations that have been examined in this work are depicted in Figure 5. All of the materials studied here responded similarly to each of the three values that were determined in the DMA tests, and the patterns of the curves were consistent across all cases. The inclusion of the filler in the nanocomposites had no important impact on the glass transition temperature. The highest storage modulus is reported for the pure PC polymer. The addition of filler overall decreases the storage modulus, indicating a weaker nanocomposites elastic behavior, compared to the unfilled PC material. All of the nanocomposites had similar storage modulus values. Only the 1 wt.% concentration nanocomposite had slightly lower storage modulus values than the remaining nanocomposites. Apart from that observation, overall, the DMA findings suggest that the introduction of the SiC additive had no significant effect on the polymer’s viscoelastic behavior.

**Figure 6:**
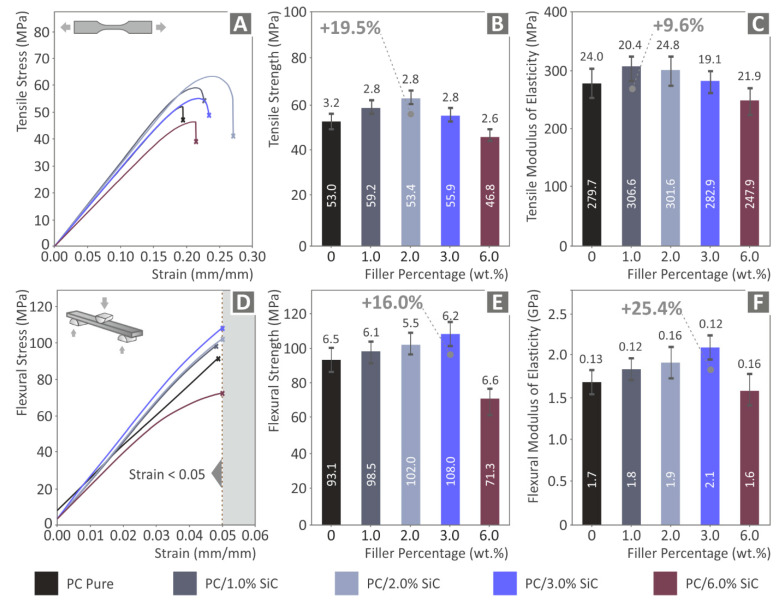
Experimental results of the tensile and the flexural tests (tests were terminated at 5% strain, in accordance with the instructions of the ASTM D790 standard): (**A**) stress compared with strain graphs, (**B**) mean calculated tensile strength and the corresponding calculated deviation out of the five samples tested (**C**) mean calculated tensile modulus of elasticity and the corresponding calculated deviation out of the five samples tested, (**D**) stress compared with strain, (**E**) mean calculated flexural strength, and the corresponding calculated deviation out of the five samples tested, and (**F**) mean calculated flexural modulus of elasticity and the corresponding calculated deviation out of the five samples tested.

The calculated tensile strength results that were acquired by the corresponding tests are presented in Figure 6. A clear enhancement of the performance of the PC polymer in the tensile test is presented, with the introduction of the SiC additive. The highest increase in the tensile strength (19.5%) is reported for the 2 wt.% nanocomposite (Figure 6B). For the SiC loadings up to 3 wt.%, the tensile strength is improved compared to the unfilled PC polymer, indicating that the study achieved its goal and SiC can enhance the strength of the PC polymer in MEX 3DP. In the 6 wt.% nanocomposite (the highest concentration studied herein), the tensile strength is reduced when comparing the experimental results with the unfilled PC polymer, indicating that a further increase in the filler loading would reach the percolation threshold of this specific additive (SiC) in the PC polymer. The use of the SiC filler has also led to an increase in the tensile modulus of elasticity (Figure 6C). The prepared nanocomposites depict a stiffer behavior than the pure PC polymer concentrations of the SiC additive up to 3 wt.%. An increase of 9.6% in the tensile modulus of elasticity was found for the nanocomposite with a 1 wt.% filler concentration. The 2 wt.% nanocomposite had a rather similar response.

The enhancement of the mechanical performance of the nanocomposites with the introduction of SiC in the PC polymer was also verified in the flexural tests, with the results presented in Figure 6D–F. A constant increase in the flexural strength of the samples is reported (Figure 6E) with the increase in the SiC concentration in the nanocomposites, for loadings up to 3 wt.%. A rather similar response is reported for the flexural modulus of elasticity (Figure 6F). An increase of 16% in the flexural strength is reported, when comparing the results with the unfilled PC thermoplastic, and the corresponding flexural modulus of elasticity is 25.4%, both with the 3 wt.% nanocomposite. In the flexural tests, similar to the tensile tests, the 6 wt.% nanocomposite exhibits lower values compared to the unfilled PC polymer.

**Figure 7:**
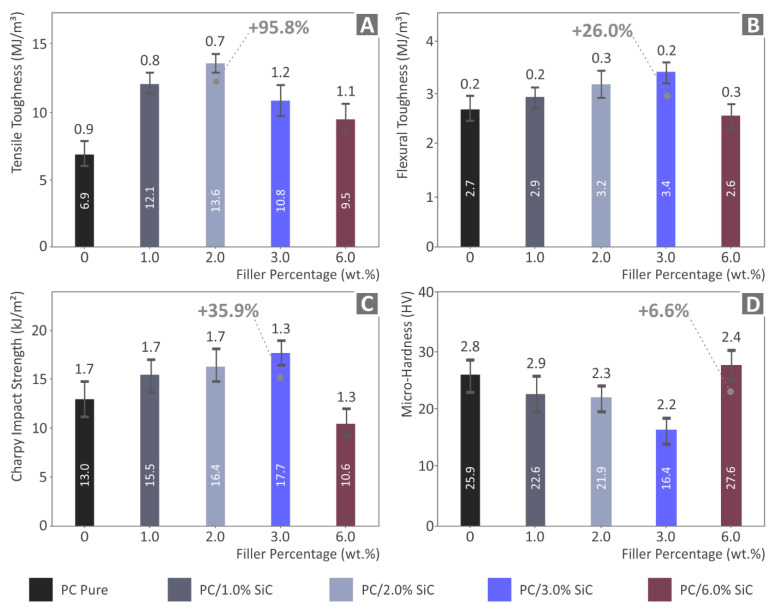
(**A**) Mean calculated tensile toughness (MJ/m^3^) and the corresponding calculated deviation of the five samples tested, (**B**) mean calculated flexural toughness (MJ/m^3^) and the corresponding calculated deviation of the five samples tested, (**C**) experimentally calculated impact strength (kJ/m^2^) and the corresponding calculated deviation of the five samples tested, and (**D**) measurements of the Vickers microhardness and the corresponding calculated deviation of the five samples tested.

From the stress compared to strain graphs, which were derived from the tensile and flexural tests, the corresponding tensile and flexural toughness values (the absorbed energy during the test) were calculated as integrals of these curves (Figure 7). These values follow the same pattern as the corresponding tests. The 2 wt.% nanocomposite exhibits the highest value for the tensile test (with a massive 95.8% increase compared to the pure PC) and 3 wt.% loading for the flexural test (25.4% improvement when correlating the results with the unfilled PC). The 6 wt.% nanocomposite in both tests (tensile and flexural) show reduced toughness values compared to the nanocomposites with lower filler concentrations, but the results, in this case, have a different pattern compared to the mechanical test results. The tensile strength of the 6 wt.% nanocomposite is lower than the unfilled PC polymer, while the tensile toughness is significantly increased when comparing the results to the unfilled PC thermoplastic. The flexural strength of the 6 wt.% nanocomposite is also lower than the unfilled PC, while the flexural toughness is about the same as the unfilled PC polymer. These differences indicate that the introduction of the additive had an impact on the energy that the material absorbed when conducting the tests and led to an increase in the strain of the material before it failed in the tests.

The impact test results follow a similar pattern to the flexural test results, with constantly increasing strength values, by increasing up to 3 wt.% of the additive concentration (Figure 7C). A 35.9% increase is reported for the 3 wt.% nanocomposite. The Vickers microhardness measurements (Figure 7D) do not have a similar pattern as the other mechanical tests. The Vickers microhardness shows a decreasing trend with the increase in the filler concentration in the nanocomposites for concentrations up to 3 wt.%. Only the 6 wt.% nanocomposite exhibited increased Vickers microhardness values, which was a 6.6% increase compared to the unfilled PC polymer.

**Figure 8:**
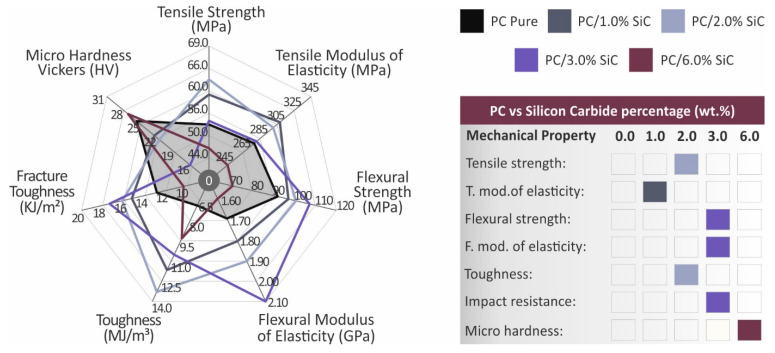
Overview of the results of the mechanical tests. The darkened region indicates the mechanical response of the unfilled PC. The material exhibiting the highest performance is indicated in the figure, on the right side.

When adding filler at loadings higher than that which exhibited the highest mechanical enhancement, a saturation of the filament in the matrix starts to occur and the mechanical properties start to decrease. The decrease in the mechanical properties is an indication of this saturation. There is a saturation threshold for the loading of each additive in each material, which was not reached in this work, as this was not within the scope of the work. Additional research is required in order to precisely calculate this saturation threshold, which can be the subject of future work. The experimental results of the mechanical tests are summarized in Figure 8. The nanocomposite exhibiting the highest response in each test is indicated.

The results that are presented herein cannot be evaluated with the existing research that is presented in the literature, since no similar nanocomposite has been developed and studied so far, for any production process. As mentioned in the introduction section, similar compounds use blends [67,68,69], therefore, the corresponding results cannot be directly correlated with the results of this work. In one study, a PC/SiC compound was examined, but no mechanical tests were conducted in the respective work [70]. The filament tensile test results of the study can only be qualitatively compared with the 3DP samples’ results, as they were not following a standard. In the filament tensile tests, enhanced mechanical behavior was determined, verifying the results of the study. However, when comparing the 3D-printed samples’ tensile response with the corresponding results for the filament, it can be observed that the tensile properties of the 1 wt.% SiC filament were the highest among all of the materials that were tested, whereas after the 3D-printing process, the highest tensile properties were achieved for the 2 wt.% SiC filled specimen. This difference can be attributed to the fact that the 3D-printed parts are highly dependent on the 3D-printing parameters. The effect of the 3D-printing parameters on the specific nanocomposites that have been developed in this work was not within the scopes of the work. One set of 3D-printing parameters was applied in order to be able to compare the results and evaluate them under the same conditions. The enhancement in the filament was measured to be smaller than the 3D-printed parts (7.6% in the filament and 19.5% in the 3D-printed parts), which also verifies this behavior. Additionally, the filament was tested under different conditions than the 3D-printed samples. The 3D-printed samples were tested according to a standard (ASTM D638), while that was not the case for the filament. Finally, due to the additional extrusion process that the material undergoes in the 3D printer head, it is possible that its properties are slightly altered in terms of the rheological characteristics and the homogenization of the additive in the matrix.

### 3.4. Examination of the Morphological Characteristics of the 3DP Specimens

**Figure 9:**
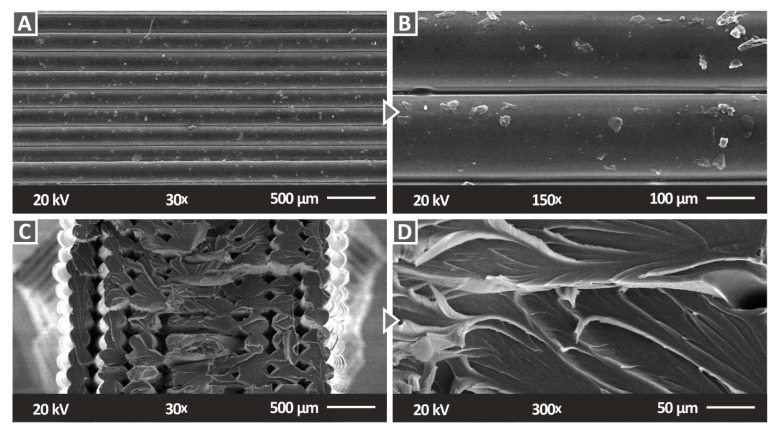
SEM micrographs of the 3D-printed sample of pure PC polymer: (**A**) 30× magnification of the side surface, (**B**) 150× magnification of the side surface, (**C**) 30× magnification of the fracture surface, and (**D**) 300× magnification of the fracture surface.

The micrographs of the pure PC polymer that were acquired by SEM are presented in Figure 9. The images of the side surface (Figure 9A,B) show an excellent 3DP quality, with defects being minimum and not affecting the structure of the part. This indicates that the 3DP settings were appropriate for the manufacturing of the part. The fracture area images of a tensile sample (Figure 9C,D) show deformation in the strands, indicating a rather ductile fracture mechanism. In the 30× magnification image, the voids that are shown are expected due to the structure of the 3DP parts, which causes porosity in them. Any voids showing filament strands that are separated can be attributed to the failure of the 3DP structure, during the experiment, which led to the failure of the part.

**Figure 10:**
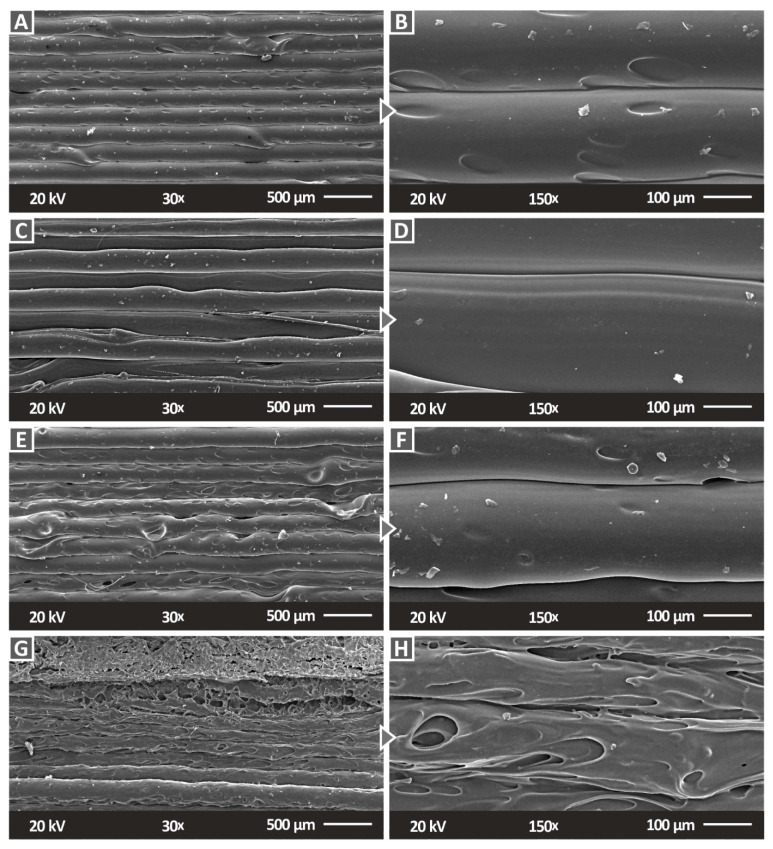
SEM micrographs of the side surface of the prepared PC/SiC nanocomposites: (**A**) 30× magnification, 1 wt.%, (**B**) 150× magnification, 1 wt.%, (**C**) 30× magnification, 2 wt.%, (**D**) 150× magnification, 2 wt.%, (**E**) 30× magnification, 3 wt.%, (**F**) 150× magnification, 3 wt.%, (**G**) 30× magnification, 6 wt.%, and (**H**) 150× magnification, 6 wt.%.

**Figure 11:**
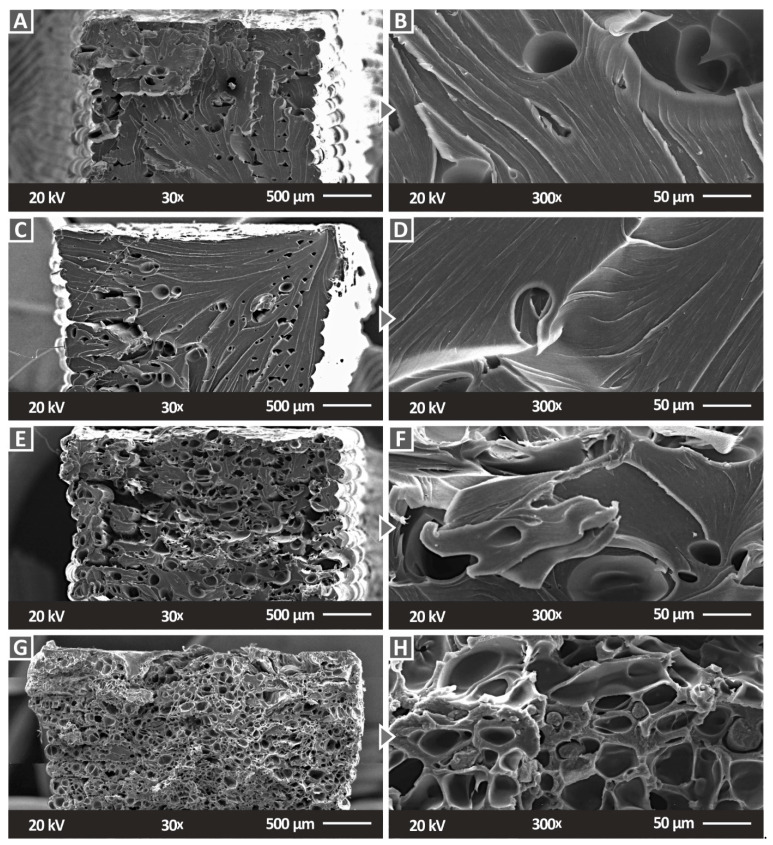
SEM micrographs of the fracture surface of the prepared PC/SiC nanocomposites: (**A**) 30× magnification, 1 wt.%, (**B**) 300× magnification, 1 wt.%, (**C**) 30× magnification, 2 wt.%, (**D**) 300× magnification, 2 wt.%, (**E**) 30× magnification, 3 wt.%, (**F**) 300× magnification, 3 wt.%, (**G**) 30× magnification, 6 wt.%, and (**H**) 300× magnification, 6 wt.%.

**Figure 12:**
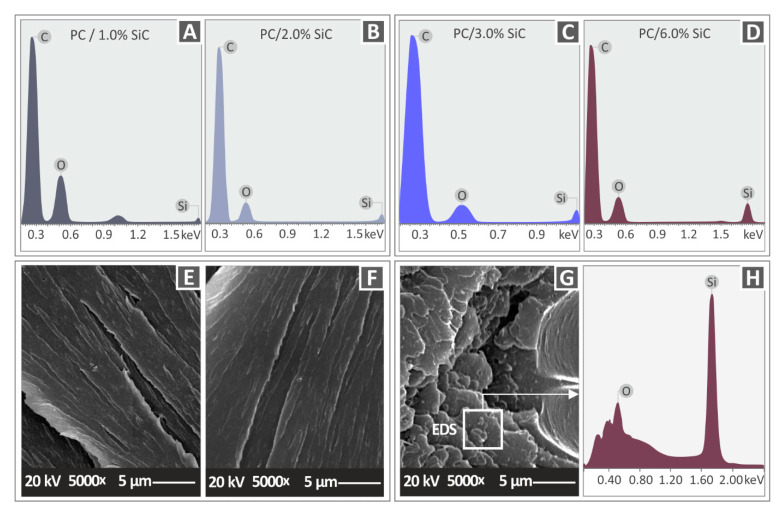
PC/SiC nanocomposite EDS graphs: (**A**) 1 wt.%, (**B**) 2 wt.%, (**C**) 3 wt.%, and (**D**) 6 wt.%. PC/SiC nanocomposites at 5000× magnification SEM micrographs of the fracture surface: (**E**) 1 wt.%, (**F**) 6 wt.%, (**G**) 3 wt.%, and (**H**) EDS graph on the area marked in Figure 12G.

Figure 10 shows the side images of the different SiC concentration nanocomposites that were prepared in this work. As the SiC concentration increases, the 3DP quality seems to be reduced, with the layer interfusion having visible defects. At the highest filler concentration of 6 wt.% (Figure 10G,H), only a few layers can be distinguished in the micrographs and the surface of the strands is not smooth. This is reflected in the mechanical performance of the 6 wt.% nanocomposite, which is decreased compared to the nanocomposites with a lower filler concentration. Moreover, the worsening of the layer fusion with the increase in the filler concentration is an indication that the material is harder to process as the filler concentration increases. Figure 11 presents the fracture surface of one randomly selected tensile test sample from each nanocomposite fabricated in the work. With the increase in the SiC concentration in the nanocomposites, the fracture area becomes rougher. In the 1 wt.% (Figure 11A,B) and the 2 wt.% (Figure 11C,D) nanocomposites, a rather brittle fracture area is observed, with minimum deformation on the part’s structure. In the nanocomposites with higher filler loadings (Figure 11E,H), internal cavities are observed and the 3DP structure is not clearly visible. This has an increasing trend with the increase in the SiC concentration in the nanocomposites. Higher magnification micrographs of 5000× (Figure 12E–G) were also taken in order to investigate the fracture areas for the agglomerations of the SiC material in the nanocomposites. No agglomerations were located in the examined areas. In the higher magnification images, Energy-dispersive X-ray spectroscopy (EDS) graphs were produced (Figure 12H), and the elements in the materials were verified. The identified elements with the EDS process were those that were expected for the materials investigated for all of the concentrations that were studied (Figure 12A–D).

## 4. Conclusions

In this study, in MEX 3DP, the effect of using SiC as an additive in the PC matrix for the enhancement of the polymer’s mechanical performance was verified. For these materials, in any production process, there are no studies of a similar nature in the literature. In this work, the impact of the concentration of the additive in the matrix material was also considered, with the 3 wt.% nanocomposite showing the highest improvement in the mechanical performance overall among the materials that were tested herein. The highest filled nanocomposite (6 wt.%) showed a reduced mechanical performance, showing that, with the process that was followed, an enhanced mechanical response can be achieved with low filler concentrations.

The nanocomposites were prepared for MEX 3DP with a thermomechanical process. The process was proven to be feasible, with any processability issues appearing at the higher filler loadings. Therefore, the process can be directly adapted to industrial-scale environments. Additionally, the thermal properties investigation verified the stability of the materials that were prepared. With this process, the inferior mechanical properties of the 3DP parts can be compensated, with the development of nanocomposites having enhanced the mechanical performance in 3DP.

Finally, this process can be characterized as cost-effective. With the process followed herein, any increase in the cost is owed to the additional cost of the SiC additive in the required raw materials. Considering that in commercial filaments the cost ratio between the raw material and the filament is about 1/10, it can be assumed that the main cost is the cost of the preparation and not the expense of sourcing the raw materials. With the methodology that has been followed here, there is a rather negligible increase in this cost, with the addition of the SiC cost, for the preparation of the nanocomposites. Considering that the 3 wt.% showed the most enhanced response, the quantities of reinforcing the materials that are required are low. The cost of the PC polymer is about EUR 0.04/gr, while the cost of the SiC additive is about EUR 0.76/gr. With these laboratory-scale prices, for the 3 wt.% nanocomposite the cost of the raw materials would be EUR 0.042/gr. This is an increase of 5% in raw materials cost. In addition, these prices can be significantly reduced in industrial-scale environments. In future work, the process can be further optimized and the required steps for its industrialization can be determined and evaluated as well.

Overall, the concluding remarks from this work are as follows:

- It is feasible to produce PC/SiC nanocomposites with a thermomechanical extrusion process;
- SiC can act as a boosting agent for the mechanical response of the PC polymer;
- Such a reinforcing process, apart from improving the mechanical properties, exploits the benefits of 3DP in fields where special demands for enhanced mechanical performance of the parts are sought;
- In MEX 3D printing overall, the 3 wt.% loading nanocomposite achieved the highest response in the mechanical tests;
- The loadings that are higher than 3 wt.% lead to inferior mechanical properties, indicating the saturation of the filler in the matrix;
- The process that was followed here is cost-effective. Only the cost of the raw materials was increased, increasing the total cost of the process for the preparation of the materials by a negligible amount;
- The process can be directly adopted for industrial use.

## Figures and Tables

**Table 1:** The Raman spectra differences in behavior (clear PC and PC/SiC nanocomposites).

Wavenumber (cm^−1^)	Assignment	Change
781	Si–O stretching	Increase of peak for SiC 6% sample [75]
966	Antisymmetric stretching of Si–O	Increase of peak for SiC samples [75]
1003	C–H bending	Increase of peak for SiC samples [73]
1175	C–O–C stretching	Increase of peak for SiC samples [73]
1344	Si–C	Increase of peak for SiC 6% sample [76]

## Data Availability

The data presented in this study are available upon request from the corresponding author.

## Supplementary Materials

The following supporting information can be downloaded at: https://www.mdpi.com/article/10.3390/polym14173492/s1, Figure S1. Settings of the 3D printing process carried out in the work and the specifications of the manufactured specimens, following the corresponding international standards. Figure S2. Nanocomposites filament quality evaluation and mechanical performance: (A) real-time filament diameter monitoring, (B) mean tensile strength results and the corresponding calculated deviation (5 samples were tested), (C) filament tensile testing, and (D) filament average tensile modulus of elasticity (5 samples were tested). Figure S3. Side surface morphology of the filament evaluated with AFM: (A) measurement of a sample in the AFM instrument, and PC (B) pure, nanocomposites with SiC (C) 1 wt.%, (D) 2 wt.%, (E) 3 wt.%, and (F) 6 wt.%.

## References

[1] Liu M., Yang S., Gao C. (2020). Scratch behavior of polycarbonate by Rockwell C diamond indenter under progressive loading. Polym. Test..

[2] Baek Y.M., Shin P.S., Kim J.H., Park H.S., DeVries K.L., Park J.M. (2020). Thermal transfer, interfacial, and mechanical properties of carbon fiber/polycarbonate-CNT composites using infrared thermography. Polym. Test..

[3] Samsudin H., Iñiguez-Franco F. (2022). Packaging and Consumer Goods. Poly(Lactic Acid): Synthesis, Structures, Properties, Processing, Applications, and End of Life.

[4] Meteab M.H., Hashim A., Rabee B.H. (2022). Controlling the Structural and Dielectric Characteristics of PS-PC/Co_2_O_3_-SiC Hybrid Nanocomposites for Nanoelectronics Applications. Silicon.

[5] Zhang W., Suhr J., Koratkar N. (2006). Carbon Nanotube/Polycarbonate Composites as Multifunctional Strain Sensors. J. Nanosci. Nanotechnol..

[6] Scardaci V., Sun Z., Wang F., Rozhin A.G., Hasan T., Hennrich F., White I.H., Milne W.I., Ferrari A.C. (2008). Carbon Nanotube Polycarbonate Composites for Ultrafast Lasers. Adv. Mater..

[7] Feltz K.P., MacFadden L.N., Gieg S.D., Lough C.P., Bezold W.A., Skelley N.W.M. (2022). Mechanical properties of 3D-printed orthopedic one-third tubular plates and cortical screws. J. 3D Print. Med..

[8] Pan K., Zhang W., Shi H., Dai M., Wei W., Liu X., Li X. (2022). Zinc Ion-crosslinked polycarbonate/heparin composite coatings for biodegradable Zn-alloy stent applications. Colloids Surf. B Biointerfaces.

[9] Lin Z.-I., Tsai H.-L., Liu G.-L., Lu X.-H., Cheng P.-W., Chi P.-L., Wang C.-K., Tsai T.-H., Wang C.-C., Yang J.H.C. (2022). Preparation of CO_2_-Based Cationic Polycarbonate/Polyacrylonitrile Nanofibers with an Optimal Fibrous Microstructure for Antibacterial Applications. Macromol. Biosci..

[10] Bagotia N., Sharma D.K. (2019). Systematic study of dynamic mechanical and thermal properties of multiwalled carbon nanotube reinforced polycarbonate/ethylene methyl acrylate nanocomposites. Polym. Test..

[11] Jorik S., Lion A., Johlitz M. (2019). Design of the novel tensile creep experimental setup, characterisation and description of the long-term creep performance of polycarbonate. Polym. Test..

[12] Pötschke P., Fornes T.D., Paul D.R. (2002). Rheological behavior of multiwalled carbon nanotube/polycarbonate composites. Polymer.

[13] Ding W., Eitan A., Fisher F.T., Chen X., Dikin D.A., Andrews R., Brinson L.C., Schadler L.S., Ruoff R.S. (2003). Direct Observation of Polymer Sheathing in Carbon Nanotube-Polycarbonate Composites. Nano Lett..

[14] Kechagias J.D., Vidakis N., Petousis M., Mountakis N. (2022). A multi-parametric process evaluation of the mechanical response of PLA in FFF 3D printing. Mater. Manuf. Process..

[15] Petousis M., Vidakis N., Mountakis N., Papadakis V., Kanellopoulou S., Gaganatsiou A., Stefanoudakis N., Kechagias J. (2022). Multifunctional Material Extrusion 3D-Printed Antibacterial Polylactic Acid (PLA) with Binary Inclusions: The Effect of Cuprous Oxide and Cellulose Nanofibers. Fibers.

[16] Vidakis N., Petousis M., Velidakis E., Tzounis L., Mountakis N., Boura O., Grammatikos S.A. (2022). Multi-functional polyamide 12 (PA12)/multiwall carbon nanotube 3D printed nanocomposites with enhanced mechanical and electrical properties. Adv. Compos. Mater..

[17] Vidakis N., Petousis M., Mountakis N., Maravelakis E., Zaoutsos S., Kechagias J.D. (2022). Mechanical response assessment of antibacterial PA12/TiO_2_ 3D printed parts: Parameters optimization through artificial neural networks modeling. Int. J. Adv. Manuf. Technol..

[18] Vidakis N., Petousis M., Velidakis E., Mountakis N., Fischer-Griffiths P.E., Grammatikos S.A., Tzounis L. (2022). Mechanical reinforcement course of 3D printed polypropylene–antimony doped Tin Oxide nanocomposites versus filler loading. Adv. Compos. Mater..

[19] Vidakis N., Petousis M., Vairis A., Savvakis K., Maniadi A. (2019). A parametric determination of bending and Charpy’s impact strength of ABS and ABS-plus fused deposition modeling specimens. Prog. Addit. Manuf..

[20] Vidakis N., Petousis M., Kourinou M., Velidakis E., Mountakis N., Fischer-Griffiths P.E., Grammatikos S., Tzounis L. (2021). Additive manufacturing of multifunctional polylactic acid (PLA)—Multiwalled carbon nanotubes (MWCNTs) nanocomposites. Nanocomposites.

[21] Tzounis L., Petousis M., Grammatikos S., Vidakis N. (2020). 3D Printed Thermoelectric Polyurethane/Multiwalled Carbon Nanotube Nanocomposites: A Novel Approach towards the Fabrication of Flexible and Stretchable Organic Thermoelectrics. Materials.

[22] Cantrell J.T., Rohde S., Damiani D., Gurnani R., DiSandro L., Anton J., Young A., Jerez A., Steinbach D., Kroese C. (2017). Experimental characterization of the mechanical properties of 3D-printed ABS and polycarbonate parts. Rapid Prototyp. J..

[23] Fang L., Yan Y., Agarwal O., Seppala J.E., Hemker K.J., Kang S.H. (2020). Processing-structure-property relationships of bisphenol-A-polycarbonate samples prepared by fused filament fabrication. Addit. Manuf..

[24] Vidakis N., Petousis M., Kechagias J.D. (2022). A comprehensive investigation of the 3D printing parameters’ effects on the mechanical response of polycarbonate in fused filament fabrication. Prog. Addit. Manuf..

[25] Koker B., Ruckdashel R., Abajorga H., Curcuru N., Pugatch M., Dunn R., Kazmer D.O., Wetzel E.D., Park J.H. (2022). Enhanced Interlayer Strength and Thermal Stability via Dual Material Filament for Material Extrusion Additive Manufacturing. Addit. Manuf..

[26] Vidakis N., Petousis M., Korlos A., Velidakis E., Mountakis N., Charou C., Myftari A. (2021). Strain Rate Sensitivity of Polycarbonate and Thermoplastic Polyurethane for Various 3D Printing Temperatures and Layer Heights. Polymers.

[27] Kattinger J., Ebinger T., Kurz R., Bonten C. (2022). Numerical simulation of the complex flow during material extrusion in fused filament fabrication. Addit. Manuf..

[28] Puttonen T., Salmi M., Partanen J. (2021). Mechanical properties and fracture characterization of additive manufacturing polyamide 12 after accelerated weathering. Polym. Test..

[29] Gupta A., Hasanov S., Fidan I. (2022). Thermal characterization of short carbon fiber reinforced high temperature polymer material produced using the fused filament fabrication process. J. Manuf. Process..

[30] Arai T., Kawaji M. (2021). Thermal performance and flow characteristics in additive manufactured polycarbonate pulsating heat pipes with Novec 7000. Appl. Therm. Eng..

[31] Vidakis N., Petousis M., Grammatikos S., Papadakis V., Korlos A., Mountakis N. (2022). High Performance Polycarbonate Nanocomposites Mechanically Boosted with Titanium Carbide in Material Extrusion Additive Manufacturing. Nanomaterials.

[32] Pandey R., Bux S., Shrivastava A., Choubey A., Singh S. (2022). A process optimization of additive layer manufacturing processes for the production of polymer composite-based components. Mater. Today Proc..

[33] Vidakis N., Petousis M., Velidakis E., Spiridaki M., Kechagias J.D. (2021). Mechanical performance of fused filament fabricated and 3d-printed polycarbonate polymer and polycarbonate/ cellulose nanofiber nanocomposites. Fibers.

[34] Farcas M.T., Stefaniak A.B., Knepp A.K., Bowers L., Mandler W.K., Kashon M., Jackson S.R., Stueckle T.A., Sisler J.D., Friend S.A. (2019). Acrylonitrile butadiene styrene (ABS) and polycarbonate (PC) filaments three-dimensional (3-D) printer emissions-induced cell toxicity. Toxicol. Lett..

[35] Hua W., Shi W., Mitchell K., Raymond L., Hua W., Shi W., Mitchell K., Raymond L., Coulter R. (2022). 3D Printing of Biodegradable Polymer Vascular Stents: A Review. Chin. J. Mech. Eng. Addit. Manuf. Front..

[36] Brognara L., Fantini M., Morellato K., Graziani G., Baldini N., Cauli O. (2022). Foot Orthosis and Sensorized House Slipper by 3D Printing. Materials.

[37] Błaszczyk M., Gabor J., Flak T., Wróbel Z., Swinarew A.S. (2022). Surgery Training System Supported by Organic Materials. Materials.

[38] Liu Z., Zhan J., Fard M., Davy J.L. (2016). Acoustic properties of a porous polycarbonate material produced by additive manufacturing. Mater. Lett..

[39] Park S.J., Lee J.E., Lee H.B., Park J., Lee N.K., Son Y., Park S.H. (2020). 3D printing of bio-based polycarbonate and its potential applications in ecofriendly indoor manufacturing. Addit. Manuf..

[40] Katoh Y., Snead L.L. (2019). Silicon carbide and its composites for nuclear applications—Historical overview. J. Nucl. Mater..

[41] Saddow S.E. (2022). Silicon Carbide Technology for Advanced Human Healthcare Applications. Micromachines.

[42] Willander M., Friesel M., Wahab Q.U., Straumal B. (2006). Silicon carbide and diamond for high temperature device applications. J. Mater. Sci. Mater. Electron..

[43] Yan W., Qin X., Zhang Z., Zhang C., Gao T. (2022). Evolution of Microstructure during Rapid Solidification of SiC under High Pressure. Adv. Condens. Matter Phys..

[44] Koyanagi T., Terrani K., Harrison S., Liu J., Katoh Y. (2021). Additive manufacturing of silicon carbide for nuclear applications. J. Nucl. Mater..

[45] Fatima A., Majid A., Haider S., Akhtar M.S., Alkhedher M. (2022). First principles study of layered silicon carbide as anode in lithium ion battery. Int. J. Quantum Chem..

[46] Dai Z., Zhou S., Yang T., Zou L. (2022). Nanofabrication of Silicon Carbide and Optical Processing and Inspection of Noncurved Mirrors. Adv. Mater. Sci. Eng..

[47] Jalluri T.D.P.V., Rao B.V., Rudraswamy B., Venkateswaran R., Sriram K.V. (2022). Optical polishing and characterization of chemical vapour deposited silicon carbide mirrors for space applications. J. Opt..

[48] Majety S., Strohauer S., Saha P., Wietschorke F., Finley J.J., Müller K., Radulaski M. (2022). Triangular Quantum Photonic Devices with Integrated Detectors in Silicon Carbide. arXiv.

[49] Castelletto S., Boretti A. (2020). Silicon carbide color centers for quantum applications. J. Phys Photonics..

[50] Liu C., Zhang Z., Si Y., Liu Y., Wang M., Lei Q. (2022). A Comprehensive Short-Circuit Protection Scheme for Series-Connected SiC MOSFETs. IEEE Open J. Power Electron..

[51] She X., Huang A.Q., Lucia O., Ozpineci B. (2017). Review of Silicon Carbide Power Devices and Their Applications. IEEE Trans. Ind. Electron..

[52] Wang F., Zhang Z. (2016). Overview of Silicon Carbide Technology: Device, converter, system, and application. CPSS Trans. Power Electron. Appl..

[53] Ariffin I.A., Kamdi Z. (2022). Review on Wear Behavior of Nickel-Silicon Carbide Electrodeposition Coating. Res. Prog. Mech. Manuf. Eng..

[54] He R., Zhou N., Zhang K., Zhang X., Zhang L., Wang W., Fang D. (2021). Progress and challenges towards additive manufacturing of SiC ceramic. J. Adv. Ceram..

[55] Liu G., Ma D., Liu H., Zhang Z., Fan C. (2022). Enhanced Effect of SiC Nanoparticles Combined with Nanohydroxyapatite Material to Stimulate Bone Regenerations in Femoral Fractures Treatment. J. Clust. Sci..

[56] Ahmed H., Abduljalil H.M., Hashim A. (2019). Analysis of Structural, Optical and Electronic Properties of Polymeric Nanocomposites/Silicon Carbide for Humidity Sensors. Trans. Electr. Electron. Mater..

[57] Guo Z., Kim T.Y., Lei K., Pereira T., Sugar J.G., Hahn H.T. (2008). Strengthening and thermal stabilization of polyurethane nanocomposites with silicon carbide nanoparticles by a surface-initiated-polymerization approach. Compos. Sci. Technol..

[58] Sternitzke M., Derby B., Brook R.J. (1998). Alumina/silicon carbide nanocomposites by hybrid polymer/powder processing: Microstructures and mechanical properties. J. Am. Ceram. Soc..

[59] Han Y., Shi X., Yang X., Guo Y., Zhang J., Kong J., Gu J. (2020). Enhanced thermal conductivities of epoxy nanocomposites via incorporating in-situ fabricated hetero-structured SiC-BNNS fillers. Compos. Sci. Technol..

[60] Thooyavan Y., Kumaraswamidhas L.A., Raj R.D.E., Binoj J.S., Mansingh B.B. (2022). Effect of combined micro and nano silicon carbide particles addition on mechanical, wear and moisture absorption features of basalt bidirectional mat/vinyl ester composites. Polym. Compos..

[61] Pelanconi M., Colombo P., Ortona A. (2021). Additive manufacturing of silicon carbide by selective laser sintering of PA12 powders and polymer infiltration and pyrolysis. J. Eur. Ceram. Soc..

[62] Grossin D., Montón A., Navarrete-Segado P., Özmen E., Urruth G., Maury F., Maury D., Frances C., Tourbin M., Lenormand P. (2021). A review of additive manufacturing of ceramics by powder bed selective laser processing (sintering/melting): Calcium phosphate, silicon carbide, zirconia, alumina, and their composites. Open Ceram..

[63] Sreenivasan R., Goel A., Bourell D.L. (2010). Sustainability issues in laser-based additive manufacturing. Phys. Procedia.

[64] Xu T.-T., Cheng S., Jin L., Zhang K., Zeng T. (2019). High-temperature flexural strength of SiC ceramics prepared by additive manufacturing. Int. J. Appl. Ceram. Technol..

[65] Polozov I., Razumov N., Masaylo D., Silin A., Lebedeva Y., Popovich A. (2020). Fabrication of Silicon Carbide Fiber-Reinforced Silicon Carbide Matrix Composites Using Binder. Materials.

[66] Du W., Singh M., Singh D. (2020). Binder jetting additive manufacturing of silicon carbide ceramics: Development of bimodal powder feedstocks by modeling and experimental methods. Ceram. Int..

[67] Raman V., Bhatia G., Bhardwaj S., Srivastva A.K., Sood K.N. (2005). Synthesis of silicon carbide nanofibers by sol-gel and polymer blend techniques. J. Mater. Sci..

[68] Alhusaiki-Alghamdi H.M. (2019). Effect of Silicon Carbide (SiC) Nanoparticles on the Spectroscopic Properties and Performance of PMMA/PC Polymer Blend. J. Mod. Phys..

[69] Lule Z.C., Kim J. (2021). Compatibilization effect of silanized SiC particles on polybutylene adipate terephthalate/polycarbonate blends. Mater. Chem. Phys..

[70] Böhning M., Goering H., Hao N., Mach R., Schönhals A. (2005). Polycarbonate/SiC nanocomposites—Influence of nanoparticle dispersion on molecular mobility and gas transport. Polym. Adv. Technol..

[71] Stuart B.H. (1996). Temperature studies of polycarbonate using Fourier transform Raman spectroscopy. Polym. Bull..

[72] Zimmerer C., Matulaitiene I., Niaura G., Reuter U., Janke A., Boldt R., Sablinskas V., Steiner G. (2019). Nondestructive characterization of the polycarbonate-octadecylamine interface by surface enhanced Raman spectroscopy. Polym. Test..

[73] Resta V., Quarta G., Lomascolo M., Maruccio L., Calcagnile L. (2015). Raman and Photoluminescence spectroscopy of polycarbonate matrices irradiated with different energy 28Si+ ions. Vacuum.

[74] Makarem M., Lee C.M., Kafle K., Huang S., Chae I., Yang H., Kubicki J.D., Kim S.H. (2019). Probing cellulose structures with vibrational spectroscopy. Cellulose.

[75] Giordano D., Russell J.K., González-García D., Bersani D., Dingwell D.B., del Negro C. (2020). Raman spectroscopy from laboratory and proximal to remote sensing: A tool for the volcanological sciences. Remote Sens..

[76] Spivak A.V., Litvin Y.A., Shushkanova A.V., Litvin V.Y., Shiryaev A.A. (2008). Diamond formation in carbonate-silicate-sulfide-carbon melts: Raman- and IR-microspectroscopy. Eur. J. Mineral..

