# Supplementary material for: Silicon Carbide Nanoparticles as a Mechanical Boosting Agent in Material Extrusion 3D-Printed Polycarbonate
Source: Polymers (Basel). 2022 Aug 26;14(17):3492. doi: 10.3390/polym14173492 (PMC9459990; doi:10.3390/polym14173492)
Supplement: Supplementary file 1 [file polymers-14-03492-s001.zip › polymers-1886326-supplementary.pdf]

Supplementary material for the article

Silicon Carbide Nanoparticles as a Mechanical Boosting Agent in Material Extrusion 3D-Printed Polycarbonate

M. Petousis [1], N. Vidakis [1,*], N. Mountakis [1], S. Grammatikos [2], V. Papadakis [3], C. N. David [4], A. Moutsopoulou [1] and S. C. Das [2]

[1] Department of Mechanical Engineering, Hellenic Mediterranean University, Heraklion 71410, Greece
[2] Laboratory for Advanced and Sustainable Engineering Materials (ASEMlab), Department of Manufacturing and Civil Engineering, Norwegian University of Science and Technology, 2815 Gjøvik, Norway
[3] Institute of Molecular Biology and Biotechnology, Foundation for Research and Technology—Hellas, Heraklion 71110, Greece
[4] Manufacturing Technology & Production Systems Laboratory, School of Engineering, International Hellenic University, Serres Campus, Serres 62124, Greece
* Correspondence: vidakis@hmu.gr; Tel.: +30-2810379227

Abstract: In this work, the effect of silicon carbide (carborundum, SiC), as a boosting agent of the mechanical response of the polycarbonate (PC) polymer, was investigated. The work aimed to fabricate nanocomposites with an improved mechanical performance and to further expand the utilization of 3D printing in fields requiring an enhanced material response. The nanocomposites were produced by a thermomechanical process in various SiC concentrations in order to evaluate the filler loading in the mechanical enhancement. The samples were 3D printed with the material extrusion (MEX) method. Their mechanical performance was characterized, following international standards, by using dynamic mechanical analysis (DMA) and tensile, flexural, and Charpy's impact tests. The microhardness of the samples was also measured. The morphological characteristics were examined, and Raman spectra revealed their structure. It was found that SiC can improve the mechanical performance of the PC thermoplastic. A 19.5% increase in the tensile strength was found for the 2 wt.% loading nanocomposite, while the 3 wt.% nanocomposite showed a 16% increase in the flexural strength and a 35.9% higher impact strength when compared to the unfilled PC. No processability issues were faced for the filler loadings that have been studied here.

S1. 3D printing parameters and geometry of the specimens manufactured according to the international standards for mechanical testing.

Parameters	Value	Units
Printing orientation	± 45	Deg.
Layer thickness	0.200	mm
Bed temperature	115	°C
Nozzle temperature	260	°C
Chamber temperature	65	°C
Number of perimeters	2	
Top solid layers	4	
Bottom solid layers	4	
Fill Density	100	%
Print speed	25	mm/s

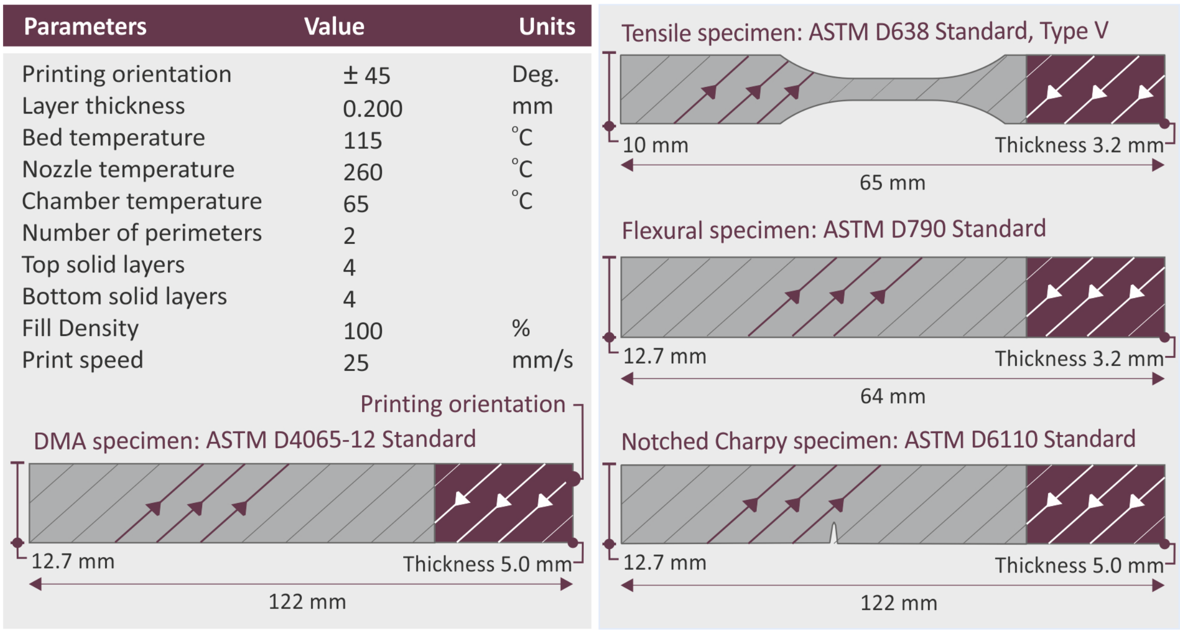

Figure S1. Settings of the 3D printing process carried out in the work and the specifications of the manufactured specimens, following the corresponding international standards.

S2. Evaluation of the nanocomposites' filament quality and mechanical performance.

Figure S2A presents filament diameter measurements taken in the extrusion process for a randomly selected time window. The filament tensile test results are presented in Figure S2B and Figure S2D. Figure S2C shows a snapshot from the filament tensile test. The experimental setup is depicted in the figure.

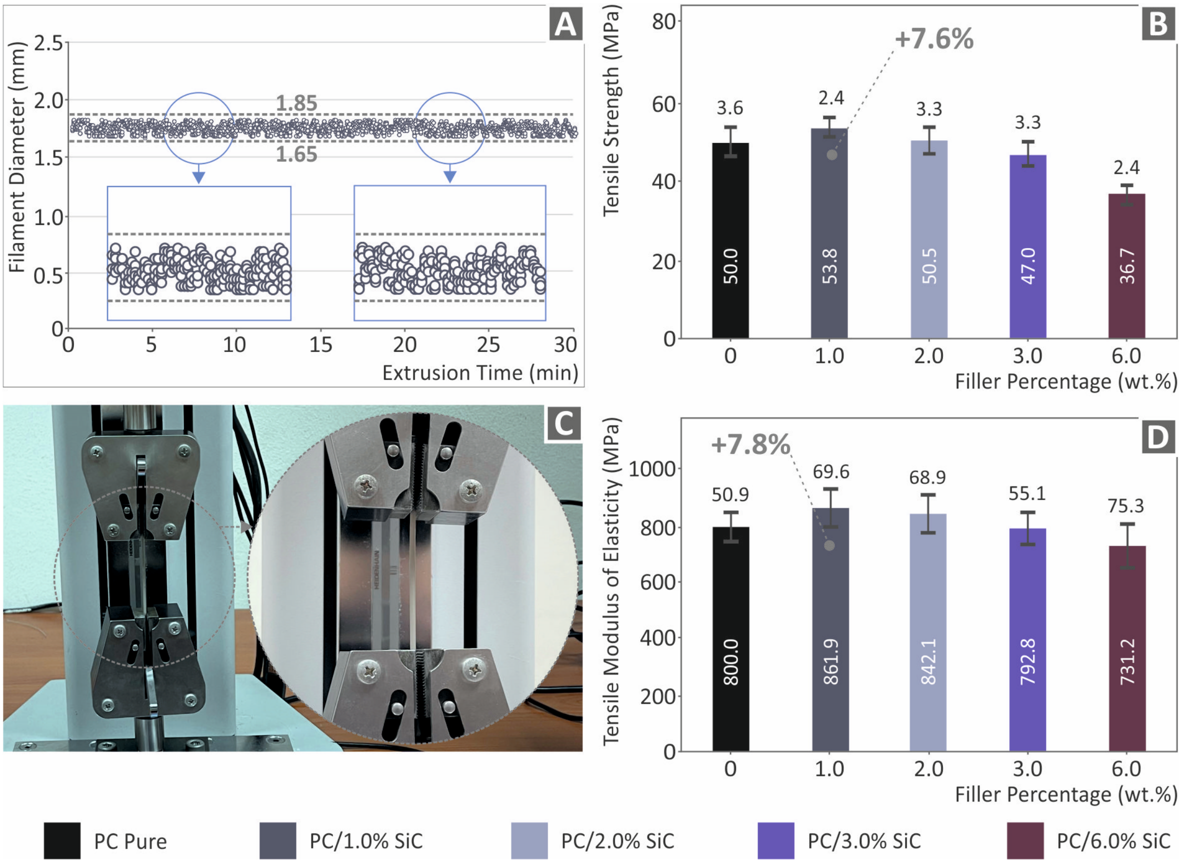

Figure S2. Nanocomposites filament quality evaluation and mechanical performance: (A) real-time filament diameter monitoring, (B) mean tensile strength results and the corresponding calculated deviation (5 samples were tested), (C) filament tensile testing, and (D) filament average tensile modulus of elasticity (5 samples were tested).

Figure S3 presents the morphological examination results on the side surface of the filament. The morphology of the side surface of the filaments produced in this work (pure PC and PC/SiC nanocomposites) was captured with Atomic Force Microscopy (AFM).

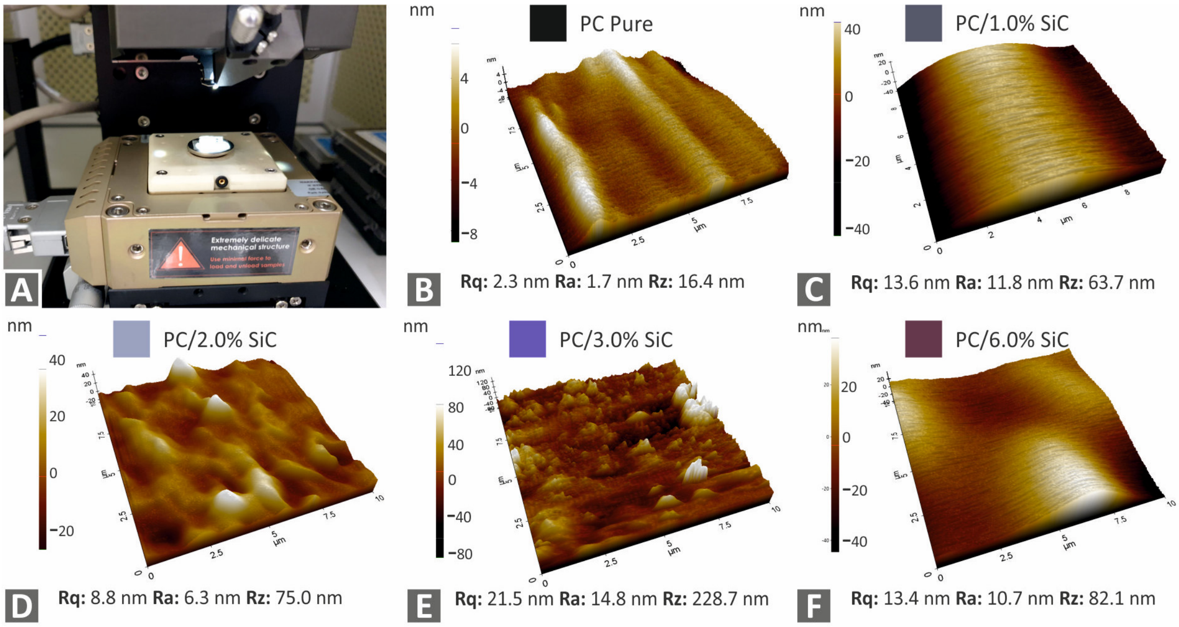

Figure S3. Side surface morphology of the filament evaluated with AFM: (A) measurement of a sample in the AFM instrument, and PC (B) pure, nanocomposites with SiC (C) 1 wt.%, (D) 2 wt.%, (E) 3 wt.%, and (F) 6 wt.%.
